# IMPROVING DEMENTIA CARE IN CO-CREATION BETWEEN SCIENCE, PRACTICE, AND EDUCATION

**DOI:** 10.1093/geroni/igac059.1423

**Published:** 2022-12-20

**Authors:** Simone de Bruin, Simone de Bruin

## Abstract

Long-term care is transitioning from a medical-somatic model of care to a psychosocial model of care. This transition requires new ways of working, and thus different competences of health and social care professionals. Innovative learning environments of multiple stakeholders in long-term care are increasingly being created in the Netherlands to foster co-creation between science, practice (including persons with dementia) and education of current and future health and social care professionals. In this symposium, we will share experiences from the Netherlands. The first presentation describes the challenges current and future health and social care professionals are facing with regard to innovation in dementia care. We will further explain how these experiences were used to set up a learning community ‘living well with dementia’. The second presentation is dedicated to a participatory action research to develop new collaboration practices to address the often under-addressed theme of meaning in life among community dwelling older adults. The third presentation will focus on a co-creation project dedicated to a care innovation for migrants with dementia, being a guideline for managing challenging behaviour at home. In the plenary discussion after the three presentations, participants are invited to share experiences from their own countries and discuss the lessons learned.

